# *Huffmanela lusitana* sp. n. (Nematoda: Trichosomoididae) infecting pouting, *Trisopterus luscus* (Teleostei: Gadidae) off the Atlantic coast of Portugal

**DOI:** 10.1016/j.ijppaw.2019.05.010

**Published:** 2019-05-30

**Authors:** Paula Ramos, Raquel Carvalho, Fernanda Rosa, Graça Alexandre-Pires, Fernanda Seixas, Alexandra Esteves, David Huffman

**Affiliations:** a Pathology Laboratory of Aquatic Animals, Portuguese Institute for the Sea and Atmosphere, I.P. (IPMA), Rua Doutor Alfredo Magalhães Ramalho, 6, 1495-165 Algés, Portugal; b Centre of Studies in Animal and Veterinary Science (CECAV), University of Trás-os-Montes e Alto Douro (UTAD), Apartado 1013, 5001-801, Vila Real, Portugal; c Instituto Superior de Agronomia, Universidade de Lisboa, Tapada da Ajuda, 1349-017 Lisboa; Centro de Estudos do Ambiente e do Mar, LA, Faculdade de Ciências, Universidade de Lisboa, Campo Grande, 1749-016 Lisboa, Portugal; d CIISA-FCT-UID/CVT/00276/2013 - Faculty of Veterinary Medicine, University of Lisbon, Avenida da Universidade Técnica, 1300-477, Lisboa, Portugal; e Department of Biology. 212 Freeman Aquatic Biology Bldg, Texas State University, San Marcos, TX, 78666, USA

**Keywords:** *Trisopterus luscus*, Dark muscle, *Huffmanela lusitana* sp. n., Nematode, Portuguese coast, Marketed infected food-fish

## Abstract

Some pouting caught off the Atlantic coast of Portugal are discarded as unmarketable due to a dark discolouration of the skin and muscle. This study investigates the cause of this condition, describes the new parasite species responsible, and highlights the importance of educating those in charge of premarket inspection of food fish in order to reduce likelihood that consumers will eat infected fish. Macroscopically, infected fish showed considerable heterogeneity in darkening of the skin and hypaxial and epaxial muscles. Microscopical observation revealed bipolar nematode eggs in varying stages of development arranged in a linear pattern along muscle fibers. Histopathology confirmed the presence of eggs of a nematode of the genus *Huffmanela* Moravec, 1987 as the cause of muscle darkening and established a relationship between infection intensity and consequent darkened appearance of the tissues. The eggs are oval or barrel-shaped, with a smooth surface and polar plugs at opposite ends. The thin outer vitelline membrane is smooth and lacks ornamentation. Under light microscopy, the main eggshell of older eggs exhibits the outermost delicate and smooth vitelline membrane, and a thicker layer, correspondent to chitinous and chondroitin proteoglycan layers. Scanning electron microscopy of eggs confirmed light microscopic studies, namely the presence of a smooth vitelline membrane surrounding the egg. Microscopic and ultrastructural characteristics of eggs, and a new host family in a new geographic area, all suggest that a new species, herein named *Huffmanela lusitana* sp. n. is involved.

## Highlights

• *Huffmanela lusitana* sp. n in *Trisopterus luscus*.
• Dark muscle due to *Huffmanela* eggs.
• *H. lusitana* sp. n as the cause of disgusting aspect of infected fish meat.
• *H. lusitana* sp. n as a zoonotical potential infection to consumers.
• *H. lusitana* sp. n. as a constraint factor of healthy wildlife stocks.

## Graphical abstract

**Figure:**
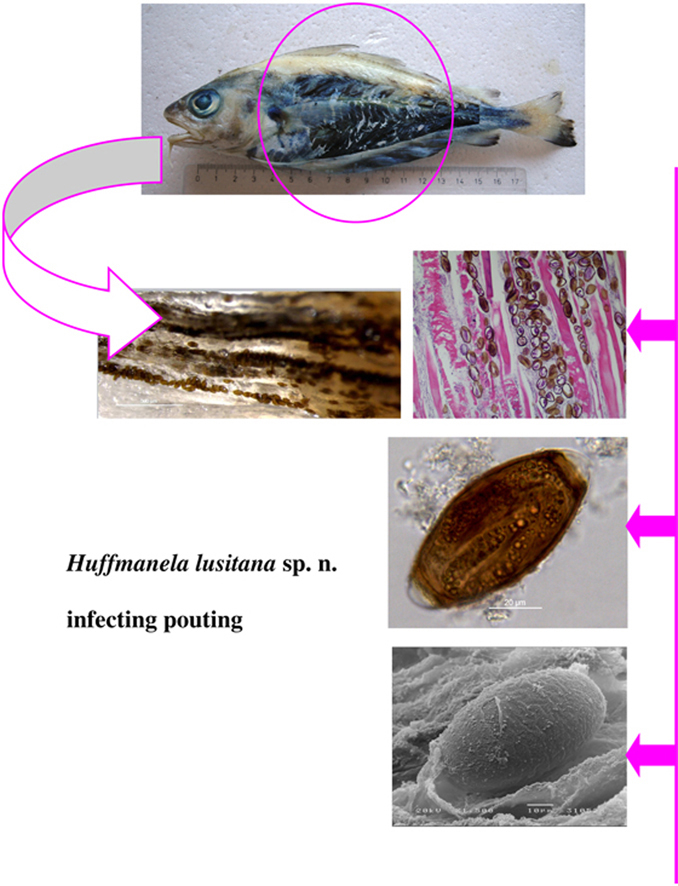

## 1 Introduction

Fish consumption in Portugal is reported as 62 kg/year/person (FAO, 2010), which makes Portugal the largest consumer of fish in the European Union (EU). Pouting, *Trisopterus luscus* (Linnaeus, 1758) (Gadiformes: Gadidae) is a common marine food fish caught off the Atlantic coast of Portugal. In the European Union, fishery workers operate under detailed rules relating to the detection of parasites in fish products destined for human consumption (Commission Regulation EC No. 2074/,2005), and the findings herein may help to provide a framework for the development of new guidelines appropriate for grading of commercially caught pouting.

In Portugal, the presence of *Huffmanela* sp. Moravec (1987) (Trichinelloidea: Trichosomoididae) nematodes infecting muscles of pouting was reported by Ramos (IPMA Report, 2002; unpublished data). This first data was presented in a scientific meeting (Mendes et al., 2005) and afterwards resulted in an MSc thesis by Mendes (2006), who described a “range of colour change” of infected pouting to support veterinarian control of fishery products. Later, this *Huffmanela* infection was revisited (Esteves et al., 2009), but the species remained unidentified. More recently, another population of *Huffmanela* sp. was discovered (Esteves et al., 2016) in a fish from a new host and family, *Microchirus azevia* (Pleuronectiformes: Soleidae) caught off the Portuguese coast, which possibly represents yet another species.

The genus *Huffmanela* Moravec, 1987 comprises 20 nominal species (Ruiz and Bullard, 2013; Ruiz et al., 2013; Justine and Iwaki, 2014) with hosts representing various fish families, but prior to our report, no *Huffmanela* population had been reported from a gadiform fish.

Most diagnosed *Huffmanela* species are classified mainly based only on morphological characteristics of their eggs, and life-cycle features such as host family and targeted tissue (Bullard et al., 2012; Ruiz et al., 2013; Justine and Iwaki, 2014). Exceptions are *H. balista* Jean-Lou Justine, 2007, *H. canadensis* Moravec et al. (2005), *H. huffmani* Moravec (1987), *H. longa* Jean-Lou Justine, 2007 and *H. moraveci* Carballo and Navone (2007), for which the adult worms have also been described.

The aim of this parasitological study of pouting caught off the Portuguese coast is to describe the new *Huffmanela* species involved and the lesions it causes in order to provide commercial food fish operators and also veterinary and food-safety authorities with new knowledge necessary to establish models for risk assessment and risk management.

## 2 Material and methods

The study was carried out from December 2011 to January 2012 at the fish auction in Figueira da Foz. Eleven specimens of pouting captured along the Atlantic Coast of Portugal between Figueira da Foz and Cabo da Roca using bottom trawls and gillnets, were selected based on their dark colour. Ten pouting were preserved in 10% neutral buffered formalin, while one was refrigerated and transported to the Pathology Laboratory of Aquatic Animals (Portuguese Institute of Sea and Atmosphere, IPMA). The study also included another refrigerated pouting without any change in skin colour for comparative purposes. Fish weight and length were measured and a database of photographs was built using a digital camera (Samsung ES15-^®^).

The intensity of darkening of the flesh was observed after removing the skin. The flesh of sample fish was graded subjectively as Grade 1 (normal colouration with no darkening), Grade 2 (with slight to moderate darkening), and Grade 3 (with intense darkening). Some muscle samples of all specimens were dehydrated in a graded series of ethanol, embedded in paraffin, sectioned at 3 μm and stained with Harris haematoxylin and eosin (H&E), according to standard methods. The number of brown eggs was counted in five random fields on histological slides from each pouting and data were recorded as number of eggs followed by the mean.

Morphological and ultrastructural studies of the eggs were performed on tissues from the refrigerated pouting. Samples of darkened musculature were examined with a stereomicroscope on wet-mounted slides without coverslip pressure in order to assess the presence of eggs, larvae or adult nematodes. The eggs (*n* = 187) were photographed and measured (length with and without plugs, and width) using a Leitz Laborlux K light microscope (LM) connected to a Leica DFC 420 camera and using the measurement software LAS (Leica Application Suite, 2009). Measurements are reported in micrometers (μm) in the form mean (standard deviation; minimum – maximum). Polar plug measurements represent the axial distance between external membrane of the embryo and the outer vitelline membrane.

For scanning electron microscopy (SEM), darkened muscle and isolated eggs were rinsed by pipetting and routinely processed. Fresh tissues with eggs were post-fixed in 2.5% glutaraldehyde in 0.1M cacodylate buffer (pH 7.4) at 4 °C (overnight) and then washed in buffer twice. The specimens were dehydrated through a graded ethanol series and dried using the critical point method. They were then sputter-coated with gold and mounted on metal stubs. SEM photographs were taken with a JEOL5200-LV electron microscope.

Eggs morphology and measurements were used to classify eggs into six presumed developmental stages from the least to the most developed as follows: Stage 1 (less developed eggs with no polar plugs, probably unfertilized); Stage 2 (clear-shelled with incipient polar plugs); Stage 3 (amber-shelled); Stage 4 (advanced brown-shelled but with no larvae); Stage 5 (advanced brown-shelled with larvae); and Stage 6 (fully developed brown-shelled with larvae near hatching).

*Huffmanela* species identification was based on the characteristics of the advanced brown-shelled eggs with larvae and their comparison with previous descriptions (Bullard et al., 2012; Ruiz et al., 2013; Justine and Iwaki, 2014).

## 3 Results

### 3.1 *Huffmanela lusitana* sp. n

**Fig. 1:**
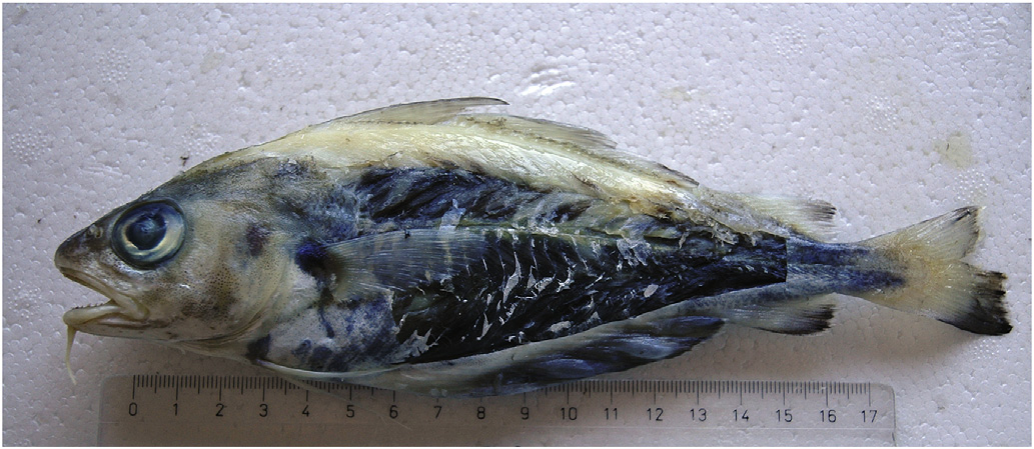
Pouting, *Trisopterus luscus* after removing the skin to show intense darkening of flesh caused by millions of very dark eggs of *Huffmanela lusitana* sp. n.

Type host-: Pouting, *Trisopterus luscus* (Linnaeus, 1758) (Fig. 1)

Site of infection: Epaxial and hypaxial musculature.

Type-locality: Atlantic coast of Portugal (between Figueira da Foz and Cabo da Roca). No available coordinates or water depth but caught by bottom trawl and gill net.

Deposition of type specimens: Syntypes, Collection of Pathology at the Portuguese Institute of Sea and Atmosphere (Lisbon, Portugal), under the code PAT/PEIXES/16/2012 to PAT/PEIXES/25A/2012 (10 infected hosts preserved in 10% neutral buffered formalin and one infected pouting refrigerated) (GBIF, https://doi.org/10.15468/x0z0xw).

Etymology: The name was related to the region where it was found and described which refers to the ancient name of the Iberian Peninsula area where the Lusitanian people lived before Roman invasions, and mainly refers to the Portugal mainland.

Adults: Unknown.

**Fig. 2:**
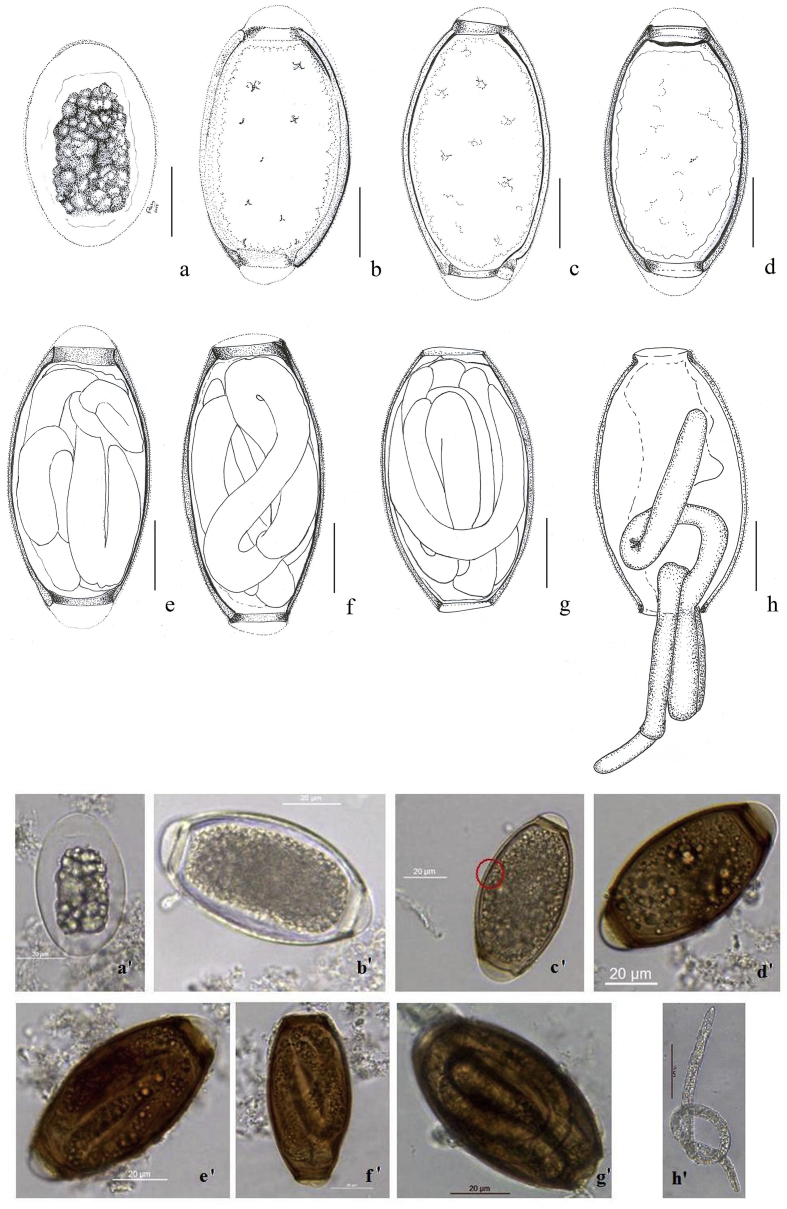
Presumptive developmental stages of eggs and larvae of *Huffmanela lusitana* sp. n. from pouting (a, a’) Stage 1: apparent unfertilized egg surrounded only by vitelline membrane with no evidence of polar plugs. (b, b’) Stage 2: clear-shelled egg with polar plugs consisting of an outer and inner layer. (c, c’) Stage 3: amber-shelled egg with bilayer eggshell observable; outer layer translucent and innermost typically dark (circle). (d, d’) Stage 4: brown-shelled egg. (e, e’, f, f’) Stage 5: advanced brown-shelled egg with larva and outer layer of polar plugs. (g, g’) Stage 6: fully developed brown-shelled egg; outer layer of polar “plugs” missing; egg apparently ready to hatch. (h, h’) Freshly hatched larva expressed from egg under coverslip pressure. (For interpretation of the references to colour in this figure legend, the reader is referred to the Web version of this article.)

Description: The eggs present the typical trichosomoidid shape, with a markedly thick eggshell surrounded by a thin, smooth, transparent vitelline membrane, closely appressed to the underlying chitinous layer. Most egg development occurred in host tissues after release from the female at an early stage and was characterized by the thickening and darkening of the shell layers, consistent changes in the polar plugs, and a consistent pattern of dimensional variation. The photos in Fig. 2 display six apparently successional stages of egg development. Measurements that follow are reported in μm and listed as Mean (SD; Min-Max).

Stage 1 eggs (apparently unfertilized) (*n* = 21, Fig. 2a, a’) contained a small central cluster of cellular material, apparently surrounded only by the vitelline membrane with no noticeable shell layers or polar plugs, and measured 48 (5.5; 39.7–59.1) in length and 30 (3.78; 22.0–36.8) in width; stage 2 eggs (*n* = 56, Fig. 2b, b’) were colourless with clear bilayered shell and well-defined polar plugs consisting of two layers, and measured 84 (3.5; 77.2–86.7) in length and 40 (1.2; 39,0–42,0) in width with polar plugs measuring 13.8; Stage 3 eggs (*n* = 6, Fig. 2c, c’) were amber-shelled and measured 79 (8.3; 65.3–86.3) in length and 39 (3.5; 33.6–42.0) in width with polar plugs measuring 14.2 (2.84; 10.8–15.1); Stage 4 eggs (*n* = 34, Fig. 2d, d’) brown-shelled with no evident larva and measured 83 (2.9; 76.1–88.3) in length and 40 (1.4; 36.9–42.9) in width, with polar plugs measuring 9.96 (1.6; 6.7–13.4); Stage 5 eggs (*n* = 50, Fig. 2e, e’- f, f’) were larvated brown-shelled and measured 75 (3.0; 69.1–82.9) in length and 42 (1.7; 36.9–45.7) in width with polar plugs smaller than in stage 4, and measuring 4.1 (1.9; 0.7–8.6); Stage 6 eggs (*n* = 20, Fig. 2g, g’) were dark brown fully developed larvae and no polar protruding plugs and measured 73 (2.6; 67.6–77.2) in length and 41 (2.4; 36.1–47.1) in width.

**Fig. 3:**
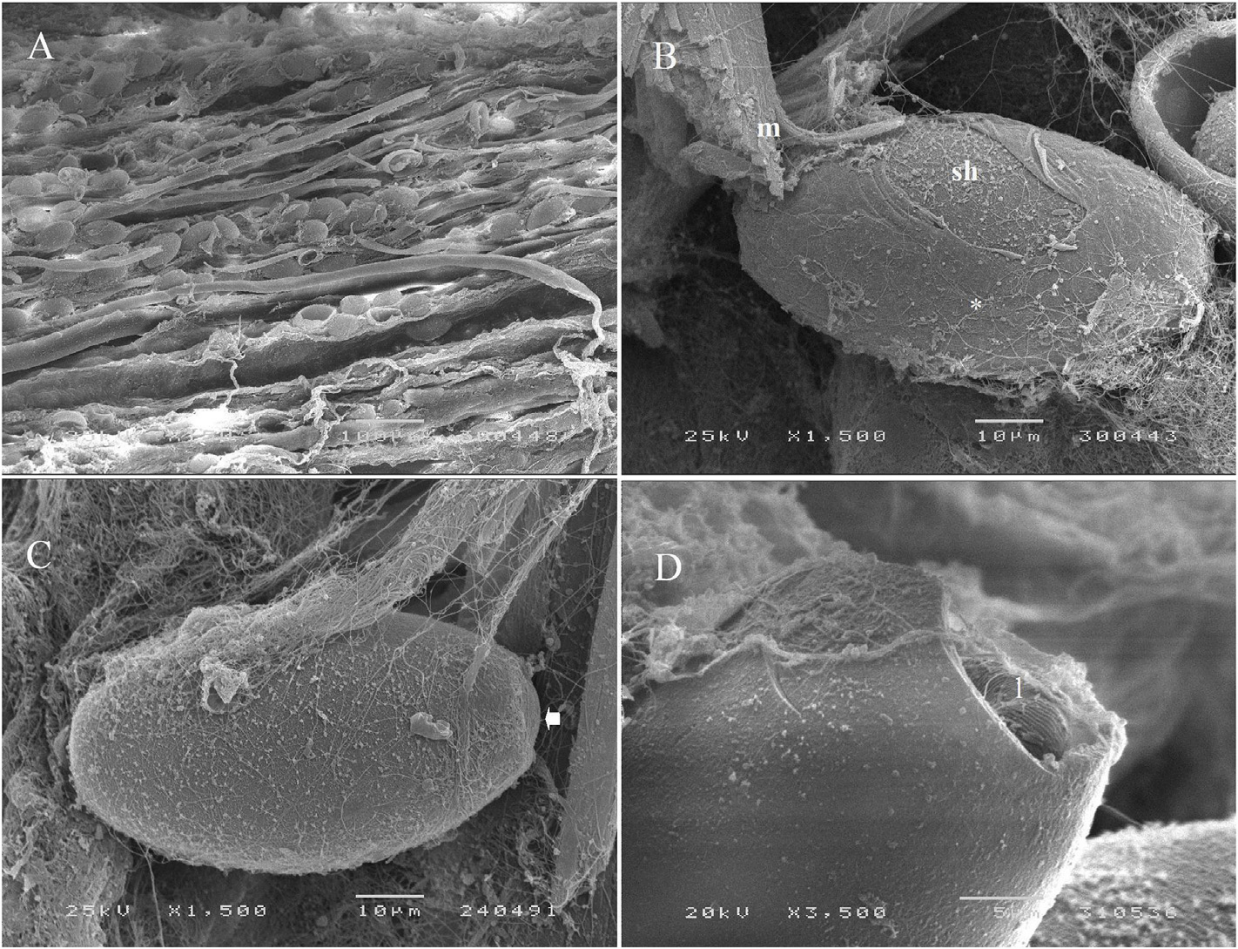
Scanning electron microscopy of *Huffmanela lusitana* sp. n. eggs in muscle of pouting. Eggs arranged in rows among muscle fibers (150x) (A); *Huffmanela* egg on the surface of the muscle fibers (m) with eggshell (sh) surrounded by a smooth vitelline membrane (*) (1 500x) (B), except at the polar plugs (arrow) (1 500x) (C); Egg partially crushed. The cuticle of the larva (l) shows regularly spaced, transverse cuticular ridges (3 500x) (D).

Eggs were oval or barrel-shaped, with a smooth surface and polar plugs. A thin and smooth vitelline membrane was present and there was no eggshell ornamentation (spines or filamentous structures). By light microscopy the main eggshell appeared bilayered; the outer layer was translucent and the inner layer was typically dark or optically dense (Fig. 2b’-c’) with a thickness ranging from 1.7 to 2.6 (amber-shelled eggs) to 2.0–3.0 (advanced brown-shelled eggs). In wet-mounted eggs, the larvae in the advanced brown-shelled eggs were tightly folded (Fig. 2g’). When subjected to coverslip pressure they emerged from one polar opening and when fully extended they were filiform and 0.37 mm in length (Fig. 2h, h’). SEM study revealed *Huffmanela* eggs arranged in rows along muscle fibres (Fig. 3a). These eggs showed a smooth vitelline membrane surrounding the eggshell (Fig. 3b and c).

The nematode larvae in the eggs show regularly spaced, transverse cuticular ridges on the body, which are clearly evident in SEM (Fig. 3d) but indistinct in LM (Fig. 2h’).

All attempts to find adult nematodes were unsuccessful.

### 3.2 Remarks on infection

**Fig. 4:**
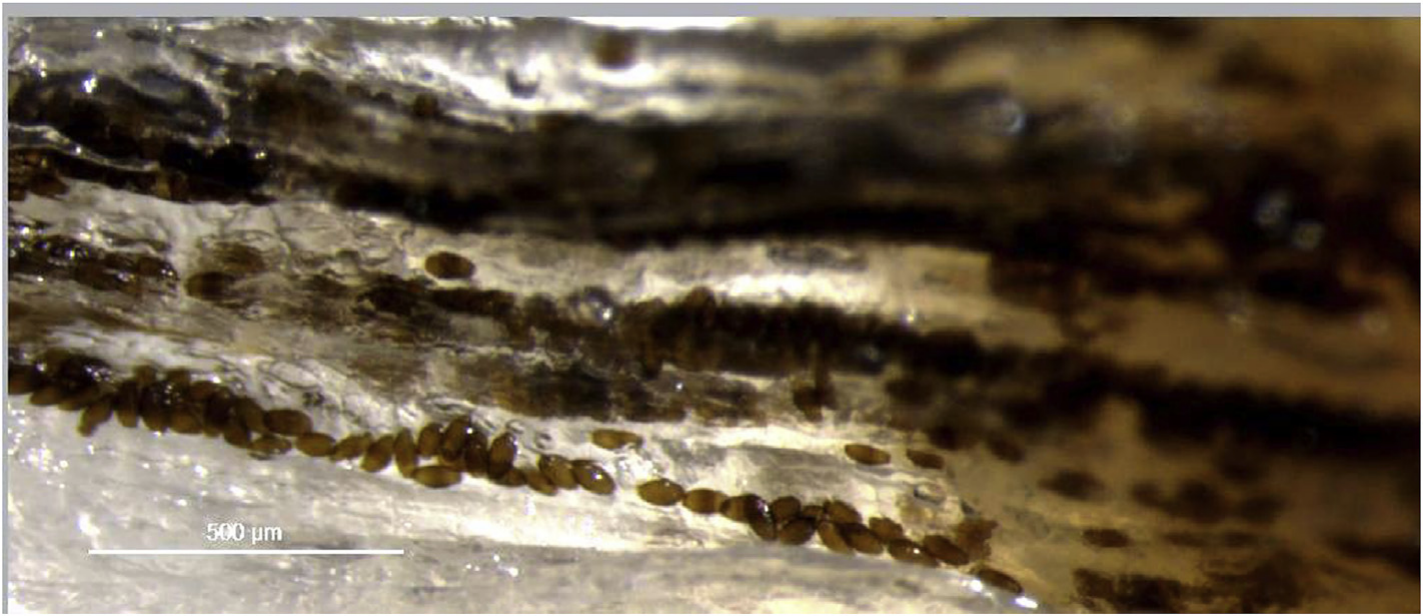
Wet mount of *H. lusitana* sp. n. eggs hypaxial muscle of pouting. The eggs are arranged in rows along muscle fibres.

The infected pouting (*n* = 10) preserved in formalin weighed (wt±SD) 137 ± 42.6 g and were 23 ± 2.7 cm in length. The corresponding values for refrigerated pouting were 230 g and 29.3 cm (Fig. 4).

After removing the skin from infected fish, we observed heterogeneous dark areas with more consistent darkness in hypaxial musculature in comparison with epaxial musculature which often had large areas with no darkened flesh. Although macroscopic examination often revealed homogenous dark patches (Fig. 1), eggs in wet mounts were arranged in rows or clusters along the muscle fibres, creating a linear pattern (Fig. 4). Eggs occurring in a group were sometimes of a uniform stage of development and sometimes of mixed stages.

**Fig. 5:**
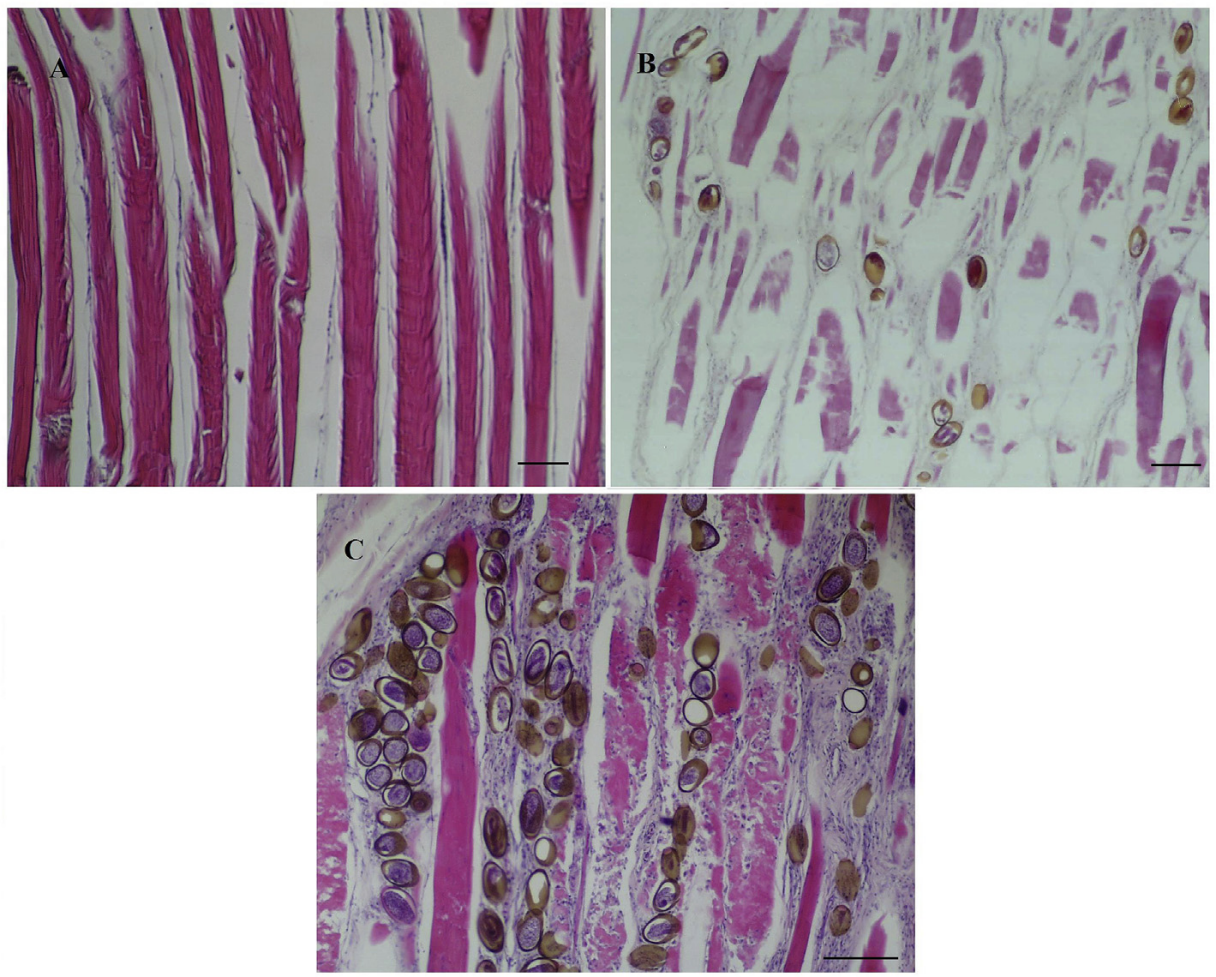
Hypaxial muscle of pouting containing eggs of *Huffmanela lusitana* sp. n. Longitudinal sections displayed at same scale and showing the relationship between darkness rating of flesh and degree of egg infection. Muscle section from fish graded as normal had 0 eggs (A); muscle section from fish graded as slight to moderate darkening had about 19 eggs (B); muscle section from fish graded as intense darkening had about 89 eggs (C). H&E. Bar: 100 μm.

**Fig. 6:**
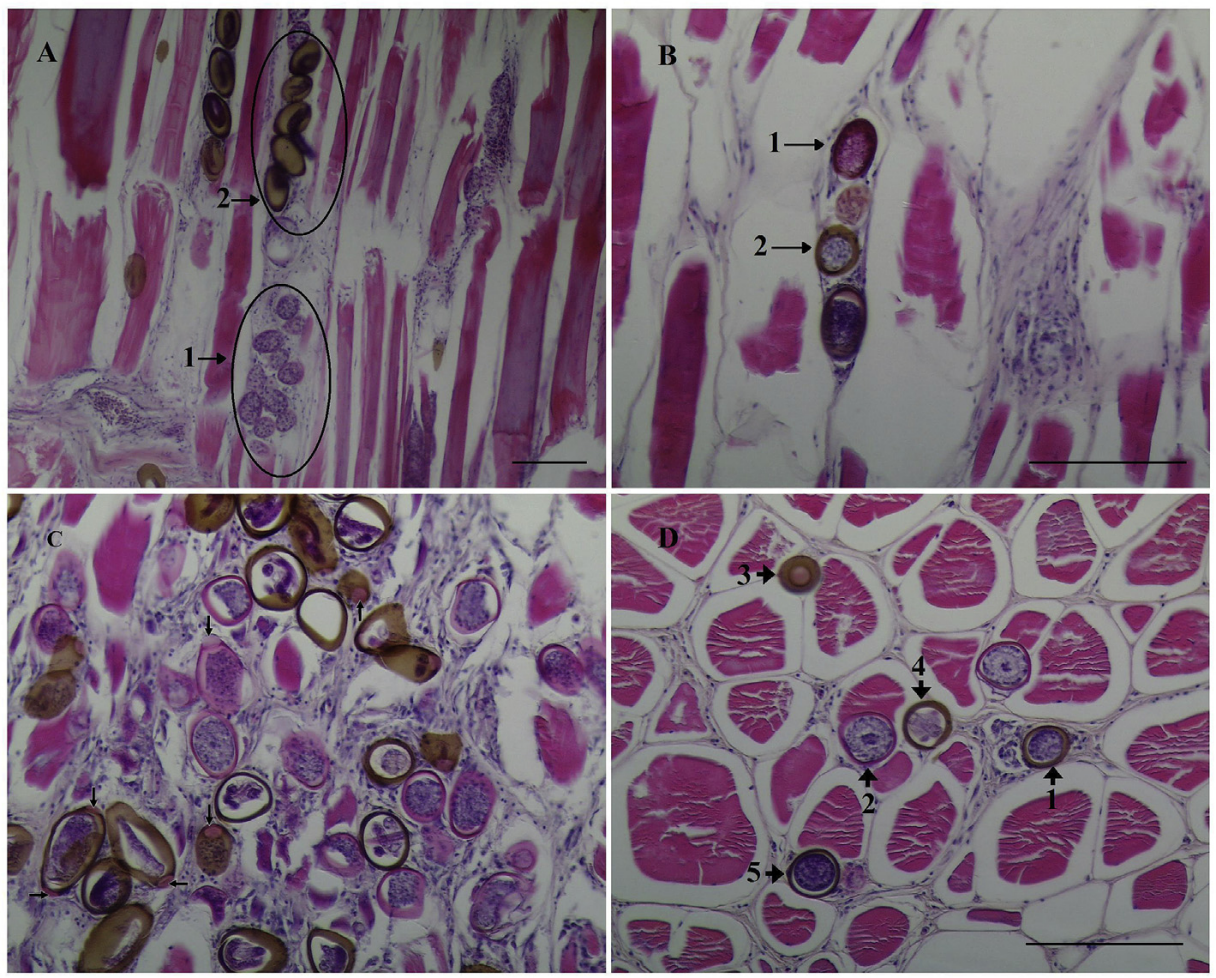
Hypaxial muscle of pouting infected with eggs of *H. lusitana* sp. n. (H&E stain). Longitudinal sections: (A) immature eggs (1) and advanced brown-shelled eggs (2) with a linear distribution; (B) clear-shelled (1) and brown-shelled eggs (2). Cross sections: (C) eggs in different stages of development and intense inflammatory lesions and muscle destruction (note the eosinophilic color of polar plugs at arrows); (D) eggs and nematodes in cross section [(1, 4, 5) eggs with intercellular location; (2) worm with intracellular location, probably 3rd or 4th stage larva; (3) apical view of a polar plug]. H&E. Bar: 100 μm. (For interpretation of the references to colour in this figure legend, the reader is referred to the Web version of this article.)

Histological sections show the presence of eggs in different stages of development, arranged linearly between muscle fibers, isolated and dispersed in muscle tissue, and also with intracellular location. Through histological observation it was possible to establish a relationship between the intensity of darkening of the muscle and the number of brown-shelled eggs in five random fields of sectioned tissue, reported as min to max (mean): Grade-1 pouting with no darkening, no eggs observed; Grade-2 pouting with slight to moderate darkening, 10 to 25 (χ = 17.5) eggs and Grade-3 pouting with intense darkening, 26 to 74 (χ = 47.6) eggs (Fig. 5). A range of development stages of eggs was observed in individual fish, from early stage clear-shelled eggs with a hyaline wall and basophilic content, to advanced, late-stage eggs with brown-shelled eggs containing basophilic larvae and with eosinophilic polar plugs (Fig. 6) in individual fish. Nematodes, probably 3rd or 4th stage larvae, were observed in some histological sections, and could be clearly seen to occur in both intercellular and intracellular locations.

**Fig. 7:**
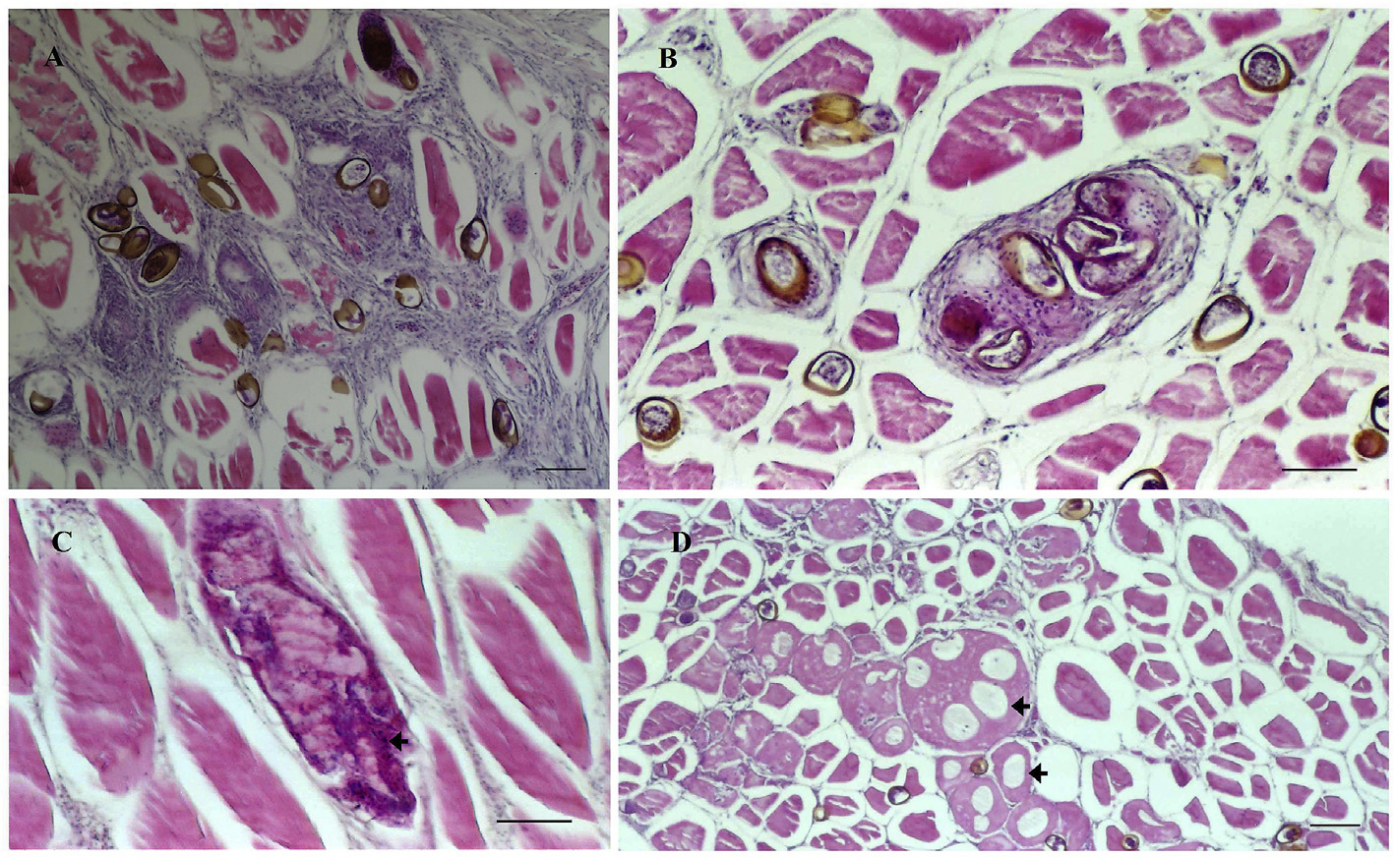
Hypaxial muscle of pouting infected with eggs of *H. lusitana* sp. n. (H&E stain). Cross sections: (A) diffuse inflammatory reaction involving eggs at different stages of development; (B) granuloma with six eggs. (C) calcification of muscle tissue (arrow); (D) degenerative changes with regular cystic structures [of presumed parasitic nature (arrows)]. H&E. Bars: A, B, D, 100 μm; C, 50 μm.

In sections of infected muscle, an inflammatory reaction and diffused multifocal granulomas involving both immature eggs (with hyaline shells) and/or brown-shelled eggs were also observed, sometimes even in fish with only slight darkening of flesh. Curiously, there seemed to be more granulomas in fish with minor darkening of flesh than in fish with heavy darkening of flesh, although this would require larger sample size to verify. No necrotic tissue was observed surrounding eggs in the granulomatous lesions. In some fields there were degenerative and dystrophic calcifications of the tissue and in others hypertrophied muscle fibres with circular and regular vacuous spaces (Fig. 7), presumed to have been previously occupied by migrating *Huffmanela* worms.

## 4 Discussion

Given that each species of *Huffmanela* deposits its eggs in a specific organ of the host (Worsham et al., 2016) and considering egg size, superficial envelope characteristics, host taxa and localities of 20 nominal species (Ruiz et al., 2013; Ruiz and Bullard, 2013; Justine and Iwaki, 2014), only four are known to infect the musculature. These are (with reported egg length and width, host taxonomy, and localities): *H*. *japonica* Moravec et al. (1998) (58–69 × 26–30 μm, Perciformes: Mullidae: *Upeneus bensasi*, Inland Sea of Japan); *H*. *shikokuensis* Moravec et al. (1998) (78–90 × 36–45 μm, Tetraodontiformes: Monacanthidae: *Stephanolepis cirrhifer*, Japan); *H*. *hamo* Justine and Iwaki (2014) (66–77 × 33–38 μm, Anguilliformes: Muraenesocidae: *Muraenesox cinereus*, Japan); and *H. banningi* Moravec (1987) (99–108 × 42–45 μm, Pleuronectiformes: Cynoglossidae: *Cynoglossus browni*, Atlantic Ocean off Senegal). To this group of muscle-infecting worms can now be added *H. lusitana* sp. n. (73–94 × 40–59 μm, Gadiformes: Gadidae: *Trisopterus luscus*, Portugal) as previously reported (Esteves et al., 2009) and described herein.

All the other named, muscle infecting *Huffmanela* species are distinct from *H. lusitana* sp. n. based on one or more of: size and morphology of eggs, host, taxonomy, infected tissue, and geographic locality. Considering measurements, the eggs from our specimens are smaller than those of *H. shikokuensis* and *H. banning*; and larger than those of *H. hamo* and *H. japonica*. With respect to morphology, in *H. banningi* the vitelline membrane is spinous, but it is smooth in *H. lusitana* sp. n. The vitelline membrane of *H. japonica* is also smooth, but the underlying eggshell presents with protuberances, while the latter is smooth in *H. lusitana* sp. n. Regarding the host species, pouting occurs in the Eastern Atlantic from the British Isles and Skagerrak to the African coast, including offshore islands, and also in the western Mediterranean (Froese and Pauly, 2014). None of the other nominal species, or even the multiple reported innominate populations, has been reported from pouting or any other gadiform, and none occurs in the Northeast Atlantic. In previous studies of *Huffmanela* cf. *lusitana* sp. n. from pouting, Esteves et al. (2009) obtained similar egg measurements (73–94 × 40–59 μm). The slight discrepancy in egg size was probably due, at least in part, to the different methods used to process studied eggs - artificial digestion in the previous study and refrigeration of the samples in this study. Thus, data obtained from morphometric, biological and ultrastructural studies of the *Huffmanela* population infecting pouting from the Atlantic coast of Portugal suggest that it represents an undescribed species.

**Fig. 8:**
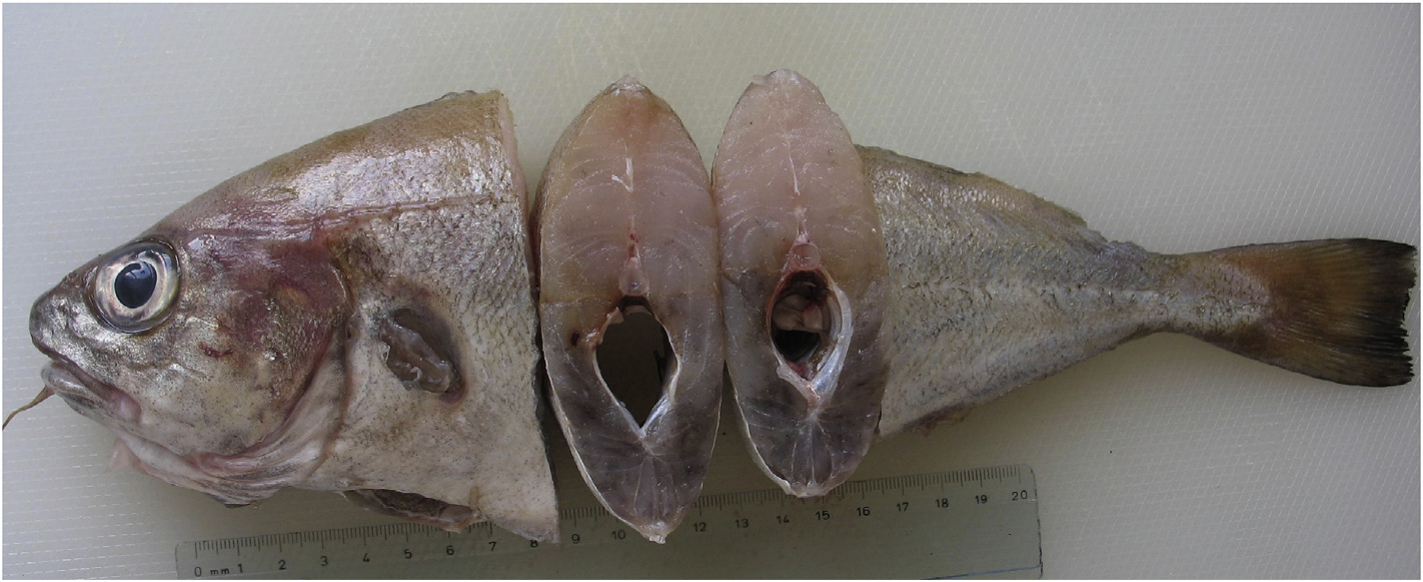
Darkened flesh of pouting rejected during preparation by end consumer.

Among the causes of fish rejection for the commercial fish market, pouting infected by *Huffmanela lusitana* sp. n. is considered negligible. Only the specimens that exhibit intense darkening of skin colour were likely to be discarded. All the infected pouting examined in this study had the recommended weight for marketing and did not reveal poor condition such as that observed by Mendes (2006), and infected specimens with slight colour change would have been cleared for human consumption. However, the skin of pouting can camouflage the presence of *Huffmanela* infections that, if passed along through the supply chain, would probably result in rejection of the flesh during preparation by the end consumer (Fig. 8). End consumers would probably see the darkened flesh as black mould, and the consumer's consequent rejection of fish as either parasitized or spoiled, will often have a long-lasting effect on that consumer's willingness to return to that market as a source of food. If the timings in the life cycle of *H. lusitana* sp. n. are similar to those revealed by Worsham et al. (2016) for *H. huffmani*, then the initial infection event of a pouting would be followed by a year or more of the larvae and adult worms wandering through the musculature of the fish before there are enough dark eggs to detect macroscopically using standard inspection protocols. Such fish in early stages of infection might not be rejected by the consumer preparing the fish for consumption, but the histological damage done by the worms migrating in and out of the muscle cells could conceivably cause the fish to be rejected on the dish due to unexpected irregularities in texture. Thus, while the rejection of darkened fish by the fishermen might seem financially insignificant, allowing fish with poor-quality flesh to move along to the consumer might have a previously unnoticed but potentially substantial depressing effect on the end market – a cost that may be difficult to quantify, but nonetheless important enough to consider.

The zoonotic impact of marketing food-fish infected with *Huffmanela* eggs is unknown. However, since all known species of *Huffmanela* are parasites of fishes (as adults) and the only known host for larvae hatched from eggs is amphipods, it is unlikely that consumption of infected pouting by humans (or any other mammal) could ever result in an infection with *Huffmanela* – although such eggs, later determined to be eggs of *Huffmanela* spp., have twice been reported in human stools after consumption of infected fish (Schouten et al., 1968; Gállego et al., 1993). With that said, it is quite likely that the rugged chitinous shells of *H. lusitana* sp. n. in infected pouting could pass unharmed through the gut of any piscivorous mammal, and that a stool examination could result in a false diagnosis of trichocephalid parasitism. So, it might be prudent to encourage medical and veterinary practitioners having patients with stools positive for bipolar eggs to inquire about recent fish consumption.

The number of eggs obtained in the different grades of colour change seems to be directly linked to the intensity of the darkening observed macroscopically, which is related to the presence of advanced brown-shelled eggs. However, pouting with slightly darkened colour presented with normal tissue interspersed with other patches having high oviposition intensity.

Macroscopically, when eggs occur in local masses in infected pouting, there is a corresponding darkening of surrounding musculature, as well as the formation of dark spots in the overlying skin. The darkened skin of infected pouting does not exhibit any peculiar pattern or lesions, in contrast to other hosts where *Huffmanela* species infect the skin. *H*. *markgracei* eggs in Atlantic sharpnose shark causes sinuous tracks occupying a swathe of the skin on the basihyal, branchial arches and the dorsal surface of the buccal cavity (Ruiz and Bullard, 2013). *H*. *oleumimica* eggs in red snapper, *Lutjanus campechanus*, are deposited in dense patches or in scribble-like tracks in the skin (Ruiz et al., 2013). Rockfish (*Sebastes* spp.) infected with *Huffmanela* eggs are associated with grossly visible skin lesions (Moravec et al., 2005). Differential diagnosis is essential to distinguish black pigmentation spots on the axillary skin of seabream, *Pagellus acarne* (Risso) from *Huffmanela* infection. The affected seabream occurs in the same geographic area as that of the studied pouting, but the diagnosis was spontaneous melanotic lesions (Ramos et al., 2013).

In wet mounts, pouting eggs are arranged in rows, probably resulting from the female dropping eggs as it moves along muscle fibres. As observed in other species - *H*. *huffmani* (Huffman and Moravec, 1988), *H*. *japonica* and *H*. *shikokuensis* (Moravec et al., 1998), *H*. c*anadensis* (Moravec et al*.*, 2005) and *H*. *paronai* (Moravec and Garibaldi, 2000) - eggs are apparently deposited in an early stage as colourless and unlarvated eggs. As the larva gradually develops, the egg grows in size and the chitinous layer becomes thicker and gradually turns from colourless to brown. This is also in agreement with the observations on *H*. *oleumimica* infection (Ruiz et al., 2013).

Eggs at different stages of development were found within the same wet mount preparation and histological field. This is probably the result of mixing of different generations of eggs, due to repeated migration of several females through the same path at different tissues (Bullard et al., 2012). Since the tenure of a laying *Huffmanela* female is apparently ephemeral (Worsham et al., 2016), the observed mixing of eggs of obviously differing ages is probably indicative of separate infection events.

The eosinophilic colour of the polar plugs contrasts with the brown coloration of the rest of the advanced brown-shelled eggs. The absence of chitin in the polar plugs explains the absence of darkening of the plugs. In the *Huffmanela huffmani* egg shell, the polar plug sits in a collar formed by the chitinous layer and consists of an electron-lucid matrix with electron-dense fibrils (Žd'árská et al., 2001).

Žd'árská et al. (2001) also referred to three layers in the *Huffmanela huffmani* egg shell: an external vitelline layer, a middle chitinous layer, and an inner “lipid” layer. More recently, Olson et al. (2012), in a comprehensive study of the egg shell of the model nematode *Caenorhabditis elegans*, determined that the third internal layer is composed, not of lipid, but of chondroitin proteoglycan, and recommended that this third layer of the nematode egg shell be referred to as the CPG layer instead of the falsely suggestive “lipid” layer. In our LM studies of the *H. lusitana* sp. n. shell, we detected the outermost delicate and smooth vitelline membrane, and a thicker layer of the shell wall, apparently correspond to the combined outer chitinous and inner CPG layers.

In the SEM image of Fig. 3B, one can clearly see where the vitelline membrane has been torn partially away to expose the smooth outer surface of the chitinous layer beneath. In the LM images of Fig. 2b’-g’, one can, with some study, discern a translucent outer layer to the wall that presumably corresponds to the chitinous layer, and a darker inner layer that presumably corresponds to the CPG layer.

During egg development of *Huffmanela lusitana* sp. n. the vitelline membrane apparently completely surrounds the egg in early stages. In Fig. 2b’-e’, the part of the membrane covering the polar plugs appears to be turgid as if filled with a clear fluid under pressure. In Fig. 2f’, the membrane over the plug appears wrinkled and flacid, and in Fig. 2g’, it appears to have ruptured or to have been partially torn away. The inner layer of the plug proper is probably still intact, since the larva has not emerged. Indeed, the SEM in Fig. 3C is of an egg with the polar section of the vitelline layer removed, exposing the core of the plug beneath. Thus, it is probably inappropriate to consider the bulging portion of the polar “plug” to be part of what actually keeps the larva in the egg. Appleton and White (1989), in a study of the polar plugs of a distant relative (*Trichuris trichiura*), observed that the bulge is apparent in LM studies, but disappears in air-dried and critical-point dried eggs, and speculated that this bulging protrusion is a “weak-spot” that may be important in initiating the hatching process.

The cuticle of a larva that was exposed when an egg was broken showed regularly spaced, transverse ridges, which were also described for larvae of *H. oleumimica* (Ruiz et al., 2013).

Muscle tissue changes and cellular responses of the host to the presence of these histozoic parasites were observed. The muscular fibrosis described in infected pouting by Esteves et al. (2009) was not observed in this study. Granulomatous reactions are sometimes formed as the host's response to the presence of persistent foreign structures. The presence of higher numbers of granulomas in fish with slight dark colour allows us to highlight the possibility that there may be parasitic granulomas in marketed fish, potentially resulting in rejection of fish on the plate due to textural irregularities. Nevertheless, the presence of cavities (of presumed parasitic origin) in muscle cells is indicative of severe cellular damage, and the areas of dystrophic calcification observed are probably indicative of prior muscle necrosis. All these alterations contribute to reduced quality of the parasitized fish as food, and may result in rejection of fish on the plate, with consequent reluctance of the consumer to return to the same market.

No encapsulated nematode bodies were found in histological sections of infected pouting inside the muscle cells, as previously reported by Esteves et al. (2009) in pouting and by Moravec et al. (1998) in *Stephanolepis cirrhifer* in Japan. It was unclear if the nematode bodies we did observe in histological sections were late larvae or adults. Moravec et al. (1998) speculated that the larvae of *H. shikokuensis* occur encapsulated in the hosts’ musculature, whereas adults, after the rupture of infected muscle cells, migrate, copulate and lay eggs in the intercellular space.

Little is known about the life-cycles of *Huffmanela* spp., although the life cycle of the only reported freshwater species, *Huffmanela huffmani* Moravec (1987), was experimentally completed by Worsham et al. (2016). Apparently, the life-cycle of *Huffmanela* species infecting internal organs may require passage through the digestive tract of a predatory fish (or death and decay of the host) before they can infect the intermediate host.

This paper describes and highlights the morphological, ultrastructural and histological features of *Huffmanela lusitana* sp. n. infection in pouting from the Atlantic Coast of Portugal, emphasizing the importance of this parasitic disease in the pouting fishery. In reality, the quantity of pouting discarded due to dark colour is negligible according to Portuguese official records, although the condition has been known since 2002 (first record observed in IPMA Laboratory). At present we continue to identify the existence of *Huffmanela* eggs in commercialized pouting. According to the Regulation 2074/2005 Annex II, Section I, Chapter I, the parasites in this study cannot be considered as “visible parasites” clearly distinguishable from the fish tissues and so, it isn't an “obviously” contaminated fish with parasites which must not be placed on the market for human consumption (Regulation 853/2004 EC. Annex III, Section VIII, Chapter V, Part D). On the other hand, the recent description of another commercialized fish infected with *Huffmanela* sp. (Esteves et al., 2016) from the same geographic area raises the question of whether or not these populations are conspecific and how important they are in the wild stocks.

## Declarations of interest

None.
